# Impact of Fluid Flow on CMOS-MEMS Resonators Oriented to Gas Sensing

**DOI:** 10.3390/s20174663

**Published:** 2020-08-19

**Authors:** Rafel Perello-Roig, Jaume Verd, Sebastià Bota, Jaume Segura

**Affiliations:** 1 Electronic Systems Group (GSE-UIB), Universitat de les Illes Balears, 07122 Palma (Balearic Islands), Spain; rafel.perello@uib.es (R.P.-R.); sebastia.bota@uib.es (S.B.); jaume.segura@uib.es (J.S.); 2 Biosensors, Medical Instrumentation and Data Analysis Group, Health Research Institute of the Balearic Islands (IdISBa), 07010 Palma, Spain

**Keywords:** MEMS resonators, VOCs, temperature sensitivity, CMOS-MEMS, gas sensors

## Abstract

Based on experimental data, this paper thoroughly investigates the impact of a gas fluid flow on the behavior of a MEMS resonator specifically oriented to gas sensing. It is demonstrated that the gas stream action itself modifies the device resonance frequency in a way that depends on the resonator clamp shape with a corresponding non-negligible impact on the gravimetric sensor resolution. Results indicate that such an effect must be accounted when designing MEMS resonators with potential applications in the detection of volatile organic compounds (VOCs). In addition, the impact of thermal perturbations was also investigated. Two types of four-anchored CMOS-MEMS plate resonators were designed and fabricated: one with straight anchors, while the other was sustained through folded flexure clamps. The mechanical structures were monolithically integrated together with an embedded readout amplifier to operate as a self-sustained fully integrated oscillator on a commercial CMOS technology, featuring low-cost batch production and easy integration. The folded flexure anchor resonator provided a flow impact reduction of 5× compared to the straight anchor resonator, while the temperature sensitivity was enhanced to −115 ppm/°C, an outstanding result compared to the −2403 ppm/°C measured for the straight anchored structure.

## 1. Introduction and Motivation

Micro/nanoelectromechanical system (M/NEMS) resonators have been widely used as mass sensors in the “More-than-Moore” paradigm because of their enhanced mass sensitivity as a result of their miniaturization down to the nanoscale level [1]. The operation principle relies on the gravimetric sensing provided by a resonant frequency down-shifting when loaded with a mass. These devices have been either operated in open- or closed-loop arrangement to obtain self-sustained oscillators capable of tracking real-time frequency shifts. Previous works based on NEMS resonators fabricated with specific technologies have reported mass sensing resolution as low as yoctograms (10^−24^ g) [2], as well as real time detection of single proteins [3]. Additionally, other works take advantage of well-established commercial technologies such as CMOS ICs to design and fabricate fully integrated CMOS-MEMS solutions; i.e., the MEMS resonator and the CMOS readout circuitry are monolithically integrated within the same die, achieving a mass resolution in the range of atto-grams (10^−18^ g) [4]. However, such an outstanding mass resolution comes at the cost of considerably small resonating structures whose effective surface for target–sensor interaction represents a limitation when sensing gas or detecting larger-sized particles. Therefore, in addition to mass sensor enhancement, distributed mass sensing is gaining increased interest given its application in volatile compound sensing. The work in [5] reported a mass resolution per unit area in the order of fg·cm^−2^ for a specific fabrication technology without integrating readout capabilities. In the same line, in a previous work, we designed and fabricated a resonant platform providing mass resolution in the order of pg·cm^−2^ by means of a CMOS-MEMS monolithic solution [6]. Other published works offer specific applications such as relative humidity measurements [7] or inkjet pico-liter droplet deposition for real-time calibration and future aerosol detection [8].

All these structures are suitable for a large number of potential applications in the biological, chemical and medical fields when coated with the proper layer materials that provide specific capabilities—for example, cancer biomarker detection [9]. The objective of adding specific coating materials is to functionalize the resonator for it to capture specific volatile organic compounds (VOCs), enabling the development of a new M/NEMS biosensors generation for the biomedical domain due to their increased effective interaction surface. Some strategies arrange several individual nanoelectromechanical sensors in an array configuration [10], while others consider larger surface resonating structures such as cantilevers [11,12], film bulk acoustic wave resonators (FBARs) [13], capacitive micromachined ultrasonic transducers (CMUTs) [14] and membrane resonators [15]. Increasing the sensing element surface improves the sensor–target interaction, and it also eases the functionalization process that requires the deposition of polymer coatings or other materials that must adhere on top of a micromachined surface. Such a deposition is performed through a variety of methods, such as airbrushing [12,16], ink jetting [17] or spin coating [13]. The accommodation of a functionalization phase into commercial technology is not a straightforward technique and, in fact, very few works combining a CMOS-MEMS resonator with a functionalization polymer are available [18].

**Figure 1:**
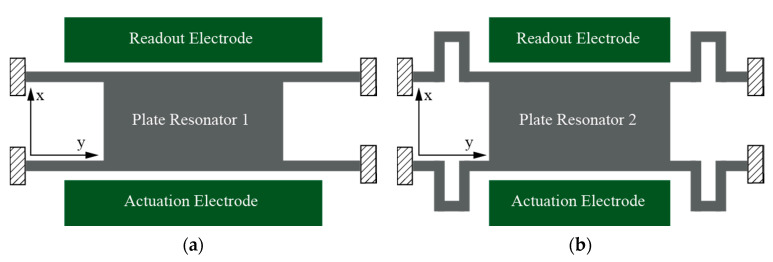
MEMS resonator schematic geometries for the fabricated and measured devices: (**a**) Plate resonator with straight anchors (PR1); (**b**) plate resonator with folded flexure anchors (PR2). Not to scale.

Miniaturized gas biosensor characterization and tuning involves experimental setups for gas mixing and delivery that require a careful analysis to account for the fundamental physical mechanism by which gas flow impacts the resonant structure behavior. Very few works detailing experimental practices for sensor calibration and operation are available. Various flow sensor designs based on MEMS resonators are available detailing that, among other effects, the well-known solid–fluid drag force impacts the resonator quality factor due to induced mechanical losses. The work in [19] characterized the change in capacitance for an in-plane capacitive MEMS sensor caused by the drag force generated from a gas stream. Another project developed a gas flowmeter based on a sail-shaped resonator working as an oscillator with a CMOS amplifier on a PCB connected to a flow chamber, obtaining a resolution as low as 0.2 mm/s [20]. Finally, a self-oscillating cantilever also proved flow sensing capabilities both in dynamic and static modes [21]. In any case, a detailed analysis of the impact of gas flow on a microelectromechanical resonator response remains an open topic. This is because the intrinsic disturbances that modify the resonator response (which occur also as a result of the specific characteristics of the resonator) when the VOC flow interacts with the sensor surface remain to be experimentally characterized in detail. In this sense, this work is focused on analyzing and evaluating the impact of a fluid flow on a MEMS plate resonator (see Figure 1a) based on analytical and experimental evidence, while designing and fabricating a new topology to diminish this impact (see Figure 1b).

This paper is organized as follows: Section 2 details the design and fabrication of the complete system, while Section 3 presents the physical mechanisms for which gas flow can modify the frequency of a micromechanical resonator. Section 4 details the setup used for the gas delivery system and presents the experimental results of flow sensitivity for open- and closed-loop topologies. Finally, Section 4 also presents the temperature sensitivity measurements and a comparison with other designs, and Section 5 summarizes the whole paper and provides the main conclusions.

## 2. Design and Fabrication

**Figure 2:**
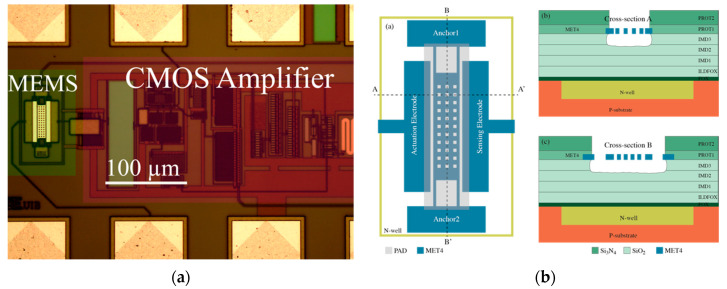
(**a**) Optical image of the overall CMOS-MEMS oscillator circuit fabricated in CMOS 0.35 μm commercial technology. (**b**) Schematic diagram showing the side-view of the MEMS resonator for two cross-sections.

The mechanical resonators used in this work were fabricated on CMOS 0.35 μm commercial technology with an additional final step performed at our lab facilities consisting of a post-CMOS mask-less wet-etch to remove the sacrificial oxide underneath the structure by using a commercial etchant; further details about the fabrication process were provided previously [22]. The technology used had four metal layers based on an aluminum composite with a TiN film; the top one was used as the resonator structural layer as it provided the larger thickness, thus resulting in a larger capacitive coupling [6]. The resonators were electrostatically actuated, while the readout scheme was capacitive, sensing the motional current generated by the resonator vibration. The whole system illustrated in Figure 2 monolithically integrates the MEMS resonator together with a CMOS readout amplifier circuit to obtain a CMOS-MEMS full-custom oscillator with quasi-digital sensor output.

**Figure 3:**
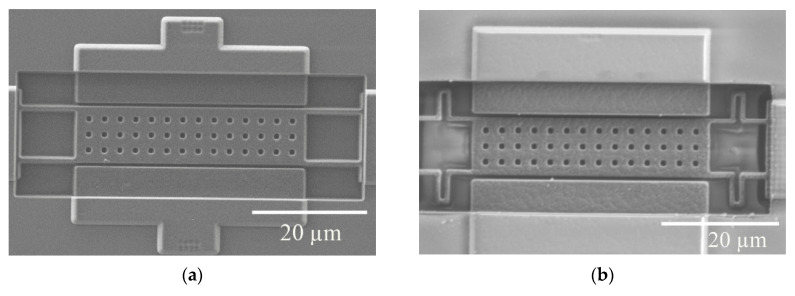
Scanning electron microscope (SEM) images for the two fabricated and tested MEMS resonators: (**a**) PR1; (**b**) PR2.

Two anchor geometries were designed, fabricated and tested. The geometric parameters for the two structures are given in Table 1. The plate resonator (PR1) depicted in Figure 3a was designed specifically for gas sensing incorporating a large sensing area that provided a high mass sensitivity per unit area [6]. A re-designed plate resonator (PR2) with the same plate area but incorporating folded flexure anchors was conceived to mitigate the impact of fluid flow effects with respect to PR1, while also reducing the temperature sensitivity (Figure 3b). Folded flexure anchors offer an additional degree of freedom displacement in the horizontal dimension (*y*-axis in Figure 1) not having its elongation constrained, which helps in improving fluid flow capabilities and temperature sensitivity. In addition to such benefits, resonator PR2 also exhibits a similar mass sensitivity per unit of area with its counterpart PR1, as indicated in Table 1. Such sensitivity ($S_{m,a}$) is computed by means of the resonance frequency ($f_{0}$), effective mass ($m_{eff}$) and platform surface (A) following Equation (1) [6].

(1) $$S_{m,a}=-\frac{2m_{eff}}{f_{0}A},$$

## 3. Theoretical Analysis

Micromechanical resonators are dramatically disturbed by a gas fluid flow induced at their surroundings, inducing an additional force on the mechanical structure that modifies its equilibrium displacement point, quality factor, etc. [23]. Achieving a practical application where the resonator works as a gravimetric sensor for VOCs requires a thoughtful understanding of the detailed interaction mechanisms between the fluid and the mechanical structure. Such analysis is key in optimizing the final sensor resolution. We analyze in detail the specific parameters that play a role in this interaction.

### 3.1. Q-Factor: Energy Losses

A typical approach describes a resonator as a one-dimension mass–spring–damper system, i.e., along the lateral direction of movement through the following equation:

(2) $$\ddot{x}+2\xi \omega_{0}\dot{x}+\omega_{0}{}^{2}x=F_{ext},$$

where $\ddot{x}$ is the acceleration, $\dot{x}$ the velocity of displacement, x the position, $\omega_{0}$ the natural frequency without damping, $F_{ext}$ the external driving force and ξ the damping coefficient. In a more convenient way, the damping coefficient is expressed in terms of the quality factor (Q), which is directly related to the movement energy losses:

(3) $$Q=\frac{1}{2\xi }.$$

Therefore, the actual resonance frequency ($f_{r}$) depends on the inverse of the squared quality factor and natural frequency ($f_{0}$), given by the structure geometrical properties [6]:

(4) $$f_{r}=f_{0}\sqrt{1-\frac{1}{2Q^{2}}},$$

For a large enough Q, the resonance frequency tends to the natural frequency. However, if Q takes smaller values, the resonance frequency depends on the damping. Therefore, it is predictable that if the resonator operates under a gas flow stream, damping rises as a consequence of an increased number of solid–fluid interactions. The final result is a resonance frequency decrease as a direct consequence of the gas flow action.

### 3.2. Drag Force

A second effect relates to the drag force ($F_{D}$) resulting from the influence of the surrounding flowing gas over the resonator. The fluid course interacts with the solid structure, inducing a resonator deflection along the gas flow direction that depends on the fluid velocity ($v_{0}$), gas density ($\rho_{g}$), section of solid exposed to the fluid flow (wl) and body geometry through drag coefficient ($C_{D}$), that can be quantified by the Reynolds number (Re) [21]:

(5) $$F_{D}=\frac{1}{2}\rho_{g}wlC_{D}v_{0}{}^{2}.$$

As a consequence, the resonator is shifted from its zero-displacement equilibrium point to a new one where the anchors stiffness equals the drag force. This causes the well-known mechanical spring-hardening effect to take place: the resonator becomes stiffer for large deflections with the consequent increase in resonance frequency. For small displacements, this effect can be approximated by a linear system where the deflection is directly proportional to the force applied, i.e., the stiffness constant (k).

(6) F=k∆x.

As the displacement increases, the resonator enters in its non-linear regime where the force is no longer only proportional to the displacement and, if the structure movement is constrained along the horizontal axis (because of the clamped-end beam anchors that induce mechanical stress), a third order term must be considered:

(7) $$F=k∆x+k_{3}{\left(∆x\right)}^{3}.$$

**Figure 4:**
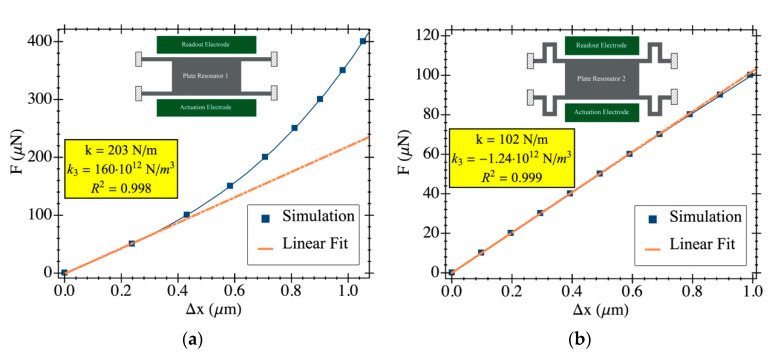
COMSOL Multiphysics simulation results depicting the stiffness constant in the *x*-axis direction and its non-linear behavior. A lateral force was applied and the resonator displacement determined. The stiffness constant and the non-linear term (k_3_) are reported in the plots. Additionally, the linear model fit for small vibration amplitude is also included for clarification; the fitting coefficient refers only to this model: (**a**) PR1 shows a deviation from the linear behavior becoming stiffer for larger vibration amplitude; (**b**) PR2 shows no spring-hardening due to the folded flexure anchors that provide free movement along the *y*-axis.

This effect was verified through COMSOL Multiphysics simulations as reported in Figure 4 where results are provided for each structure. In the simulations, a lateral distributed force was applied and the net displacement was plotted. On the one hand, PR1 (Figure 4a) shows a strong increase in its stiffness constant as it moves towards larger deflections, while PR2 (Figure 4b) shows no spring-hardening even for very large displacement due to its folded flexure anchors. In any case, even a spring-softening effect was observed (the constant k_3_ was negative) at a much smaller magnitude compared to PR1.

Therefore, the structure deflection produced by the fluid drag force can increase the resonator resonance frequency by means of the above-mentioned spring-hardening effect. In fact, this result is not only present for static deflection when the resonator vibrates around a non-zero equilibrium point, but also for dynamic operation: if the oscillation amplitude is large enough, for example in vacuum, the equivalent stiffness constant increases and so does the resonance frequency.

### 3.3. Non-Linearities: Mechanical Spring-Hardening vs. Electrical Spring-Softening

Electrical spring-softening is a resonance frequency decrease caused by an increase in the DC biasing voltage applied to the resonator for its excitation [24]. Being an attractive interaction, the electrical force driving the resonator pulls the structure further towards the electrodes along the movement direction resulting in a reduction in the mechanical stiffness constant and the resonance frequency in the following way:

(8) $$f_{r}=f_{0}\sqrt{1-\frac{V_{dc}{}^{2}C_{0}}{ks^{2}}},$$

where $V_{dc}$ is the biasing voltage, $C_{0}$ the coupling capacitance and s the resonator–driver gap. This effect is present in any electrostatic driven resonator independently of the excitation voltage, i.e., either for large or small vibration amplitudes, an increase in the polarization voltage induces a reduction in the resonance frequency. Additionally, an equivalent phenomenon can be accounted for when, having the same DC bias, the resonator deflection is large enough to experience a noticeable non-linearity in the electrostatic force so that the open-loop resonance peak bends towards smaller frequencies. While both spring-hardening and softening might happen concurrently, one tends to dominate over the other depending on various factors like resonator geometry and environmental physical and electrical parameters [25]. In our case the results shown in Figure 4, suggest that the dominant effect for PR2 was electrical spring-softening, while PR1 experienced a frequency shift towards larger values due to mechanical spring-hardening for large vibration amplitude.

## 4. Experimental Setup and Results

**Figure 5:**
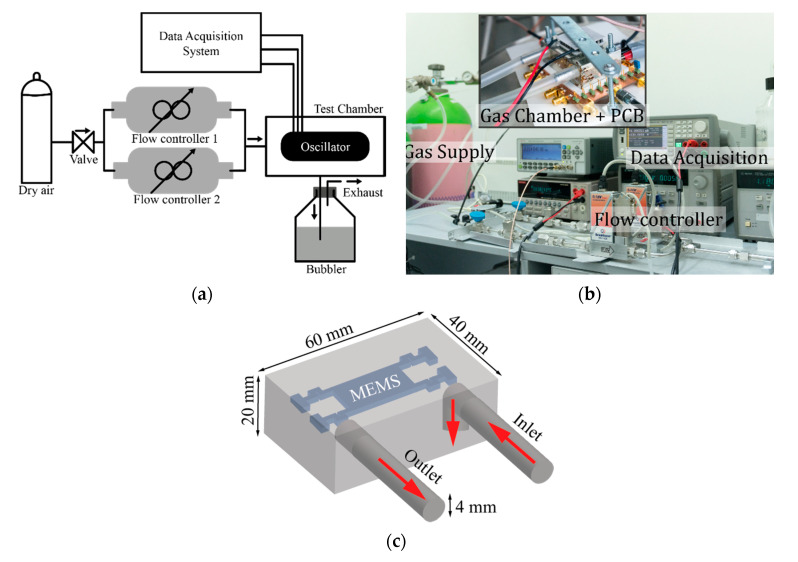
Experimental setup for gas flow test on CMOS-MEMS resonators: (**a**) schematic representation; (**b**) picture of the actual setup including the test equipment for system data acquisition; (**c**) diagram showing the gas chamber dimensions and the relative position of the fluid flow over the MEMS resonator.

Most experiments involving gas management use a calibrated source that is further mixed with a carrier to modify the gas concentration driven to the sensor being characterized. Other techniques take a solution of the desired analyte through which the gas is passed getting saturated by the analyte. Next, further mixtures can be performed to obtain the desired concentration. Most of the works published in the literature [12,16,18,26,27,28,29,30] use a similar gas injection system as the one depicted in Figure 5 to characterize resonator-based gas sensor. To the best of our knowledge, a detailed investigation into the impact of the gas flow phenomena (without considering any analyte–sensor adsorption nor chemical interaction) on the vibrating mechanical structure is not available. To conduct such an experiment and provide a systematic way to properly characterize resonator-based VOC sensor structures, we ran our experiments using dry air constituting an inert gas to guarantee minimum chemical interaction with our sensor structures. We designed and fabricated a gas chamber, whose schematic and dimensions are depicted in Figure 5c, that minimized the fluid flow direct impact over the plate resonator. This was accomplished by directing the inlet flow towards a lateral wall instead to the resonator itself.

The CMOS-MEMS resonators were characterized by both open- and closed-loop self-excited configurations to obtain an accurate description of the various effects induced by the gas flow. The flow tests were implemented using the experimental setup shown in Figure 5 that consisted of a gas supply (dry air) that ran through two digitally controlled mass flow Bronkhorst El-Flow valves to investigate the impact of gas mixture transients. The two valve outputs were mixed and fed into a sealed test chamber, where the resonator was electrically driven and measured while being kept at constant temperature. A gas outlet from the chamber was driven into a bubbler to avoid backpressure having the whole system continuously monitored by a computer. The open-loop tests were performed with a Keysight E5061B Vector Network Analyzer (VNA) to obtain the electromechanical transmission coefficient, while the frequency real-time tracking when operating as a self-sustained oscillator was done by means of a Frequency Counter Pendulum CNT-91.

### 4.1. Impact of Gas Flow

**Figure 6:**
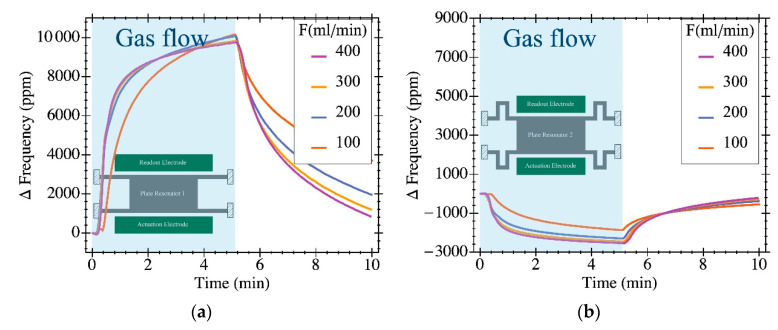
Relative frequency changes over time for a valve aperture and subsequent closure at different gas flow rates (see legend). Each of the resonators exhibits a singular response as a consequence of their different anchor geometries: (**a**) PR1 presents a much larger frequency variation, as high a 10,000 ppm, due to its clamped ends; (**b**) PR2, shows a much smaller absolute frequency change (2500 ppm) thanks to the folded flexure anchors that provide free movement along the *y*-axis; moreover, its frequency variation takes the opposite direction than for PR1.

In the first analysis, we injected various flow rates to our two resonant structures to determine the impact of such flow on the resonant frequency. Figure 6 plots the experimental results obtained when the gas flow ranged between 100 mL·min^−1^ and 400 mL·min^−1^ in steps of 100 mL·min^−1^. The minute-range transient response obtained was clearly attributed to the gas flow stationary stabilization into the chamber since the resonator response to an external disturbance takes a few seconds given its micrometric-scale dimensions. Data show a dissimilar response for the two structures both quantitatively and qualitatively. The PR1 structure resonant frequency increased while the gas flow was injected (light blue background portion of the graph) and restored to its initial value once the gas flow was removed with a relative frequency variation of 1%. However, the PR2 structure, with the folded flexure anchors, experienced a frequency variation in the opposite direction (decreasing) with respect to PR1 exhibiting a much smaller (below 0.3%) overall variation. These results suggest that the relative dominance of the non-linear spring-hardening and softening counterbalance in a considerably different manner for these structures due to the shape of the anchors.

**Figure 7:**
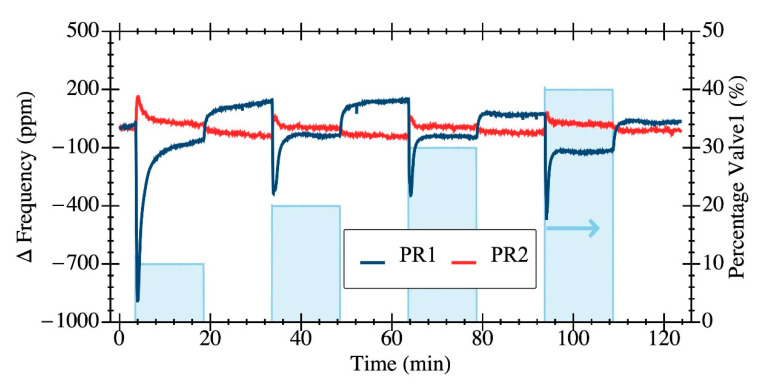
Time evolution of relative frequency change measured at the self-sustained oscillator output plate resonators: PR1 (blue line) and PR2 (red line) for a total dry air flow of 300 mL·min^−1^. Two valves were complementary switching their relative aperture (the % of valve 1 is depicted in light blue) to test the impact of an overall flow transient spike on the resonator frequency.

In the second experiment, we investigated the impact of gas flow-relative transients on the resonator response. We programed an overall constant mass flow of 300 mL·min^−1^ while varying the relative aperture of mixing valves valve 1 and valve 2 while passing dry air coming from the same source. Figure 7 reports the experimental data obtained for the resonant frequency of PR1 and PR2 structures when valve 1′s aperture was toggled from closed state to 10%, 20%, 30% and 40% aperture (valve 2 was settled to (100%—valve 1) aperture to guarantee the overall constant mass flow). Results show that PR1′s structure exhibited a relatively larger transient flow sensitivity with sharp frequency shifts followed by a transient recovery period. PR2′s structure exhibited a much lower transient flow sensitivity, thus being less impacted by gas flow transients. The perturbations induced by the gas transients do impact the resolution limit for these structures when used as gravimetric sensors since such frequency fluctuations are not caused by mass deposition or overall flow-rate variation. This means that the limit of detection for such sensors depends not only on the structure itself, but also on the gas injection method. In this sense, results in Figure 7 show that the PR2 structure is much less sensitive to these undesired transient effects, with a 10× reduction in frequency shift representing, a priori, a better structure for gravimetric-based VOC sensing.

### 4.2. Impact of Operating Pressure

**Figure 8:**
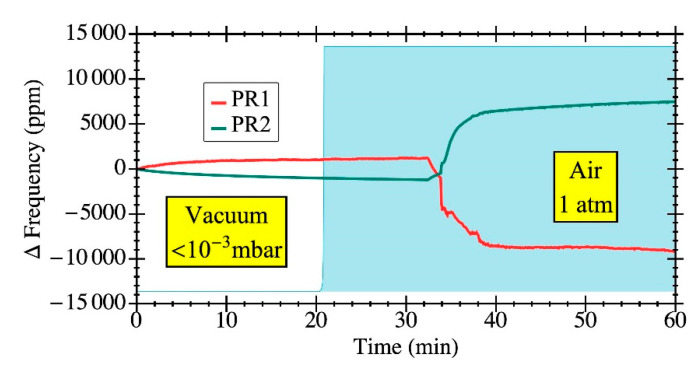
Relative frequency change for each one of the geometries due to variation in the operating pressure down to 10^−3^ mbar. The open-loop response in vacuum and air was also measured for an input power of −30 dBm so as to obtain the value of Q by curve fitting. PR1 showed a Q of 190 in air and 910 in vacuum, while PR2 reached a Q of 170 in air and 750 in vacuum.

To determine which of the mechanisms described in Section 3 dominates the frequency variations observed, we ran an additional test in vacuum conditions since the pressure at which the resonator operates impacts its resonant frequency through the variation of the quality factor Q as stated in Section 3.1 Figure 8 shows the relative frequency variation of the CMOS-MEMS oscillators when changing the operating pressure from high vacuum (<10^−3^ mbar) to ambient air pressure. It is shown that PR1 exhibits a frequency variation in the opposite direction than the one measured for PR2. The frequency change in the opposite direction for both structures is consistent with the results shown in Figure 6 where the influence of a fluid flow shifts the resonance frequency of PR1 to higher values, while in the case of PR2 it moves to lower frequencies.

If these divergences were to be caused only by a change in Q value, both structures would present a frequency shift in the same direction; i.e., when toggling from air to vacuum conditions, the frequency change should point towards larger resonance frequency for both resonators given the increased value of Q (see Figure 8) according to Equation (4). This is not the case for PR2 as it experiences a downshift of the resonant frequency due to the dominating effect of the spring-softening effect as stated in Section 3.3 As for the PR1 structure, its frequency shift when changing from vacuum to normal air pressure conditions is in the order of 10^4^ ppm (as shown in Figure 8). According to Equation (4), such a frequency shift would require a value of Q ≈ 5 in air, while the actual value derived from experimental data when fitting the open-loop response is Q = 190. This indicates, as stated in Section 3.3, that PR1 with clamped anchors experiences mechanical spring-hardening, and that the primary cause for its frequency increase is due to an increase in its oscillation amplitude up to the non-linear regime caused by a larger value of Q as a result of the vacuum conditions.

**Figure 9:**
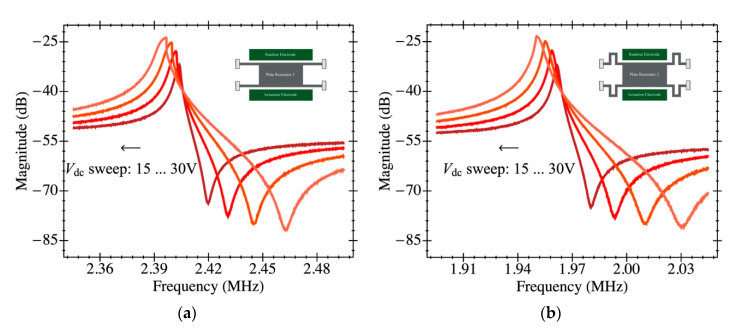
Electromechanical system transmission coefficient measured in open-loop configuration under vacuum pressure (<10^−3^ mbar) and 2 dBm input power. The amplifier gain has been reduced by increasing the capacitance at the sensing node to visualize the non-linear behavior previous to amplifier saturation: (**a**) PR1 has a biasing voltage that changes from 15 V to 30 V; (**b**) PR2 has also a sweep in the polarization from 15 V to 30 V.

To further verify these dependencies, we ran the open-loop experiments shown in Figure 9 where we measured the system transmission coefficient for a large enough input power (2 dBm). Such measurements visualize the well-known non-linear frequency shift already described, being in full agreement with the self-oscillation test.

**Figure 10:**
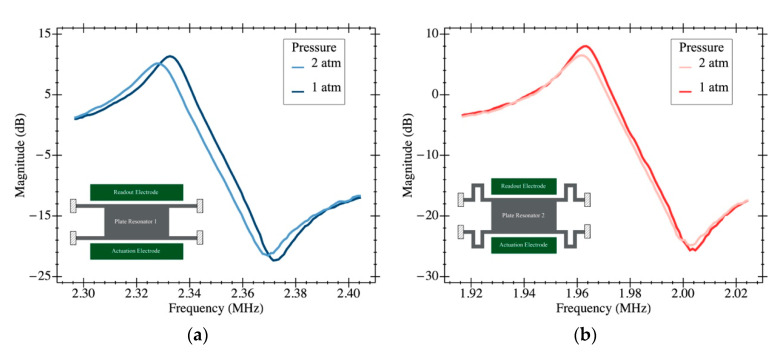
Magnitude plot of the system transmission coefficient in ambient pressure and overpressure (2 atm) operation with a DC biasing voltage of 30 V for each resonator: (**a**) PR1; (**b**) PR2. When increasing the operating pressure, the quality factor decreases and, as a consequence, so does the resonance frequency.

We ran an additional experiment to determine the impact of pressure increase on each structure. Figure 10 reports the open-loop behavior for both structures measured at 1 atm and 2 atm environments, showing the same tendency for the two resonator types—a frequency decrease with increasing pressure. Such a decrease is caused by a Q factor degradation with increase in pressure for both structures. These results indicate that the behavior observed for the experiments shown in Figure 6 and Figure 7 is not caused by a relative pressure increase induced by the fluid flow, as overpressure impacts the frequency shift of both structures in the same direction. This dissimilar behavior shown in Figure 6 and Figure 7 is due to the interaction of the folded flexure anchors with the environment fluid, mitigating the vibration losses.

### 4.3. Temperature Sensitivity

**Figure 11:**
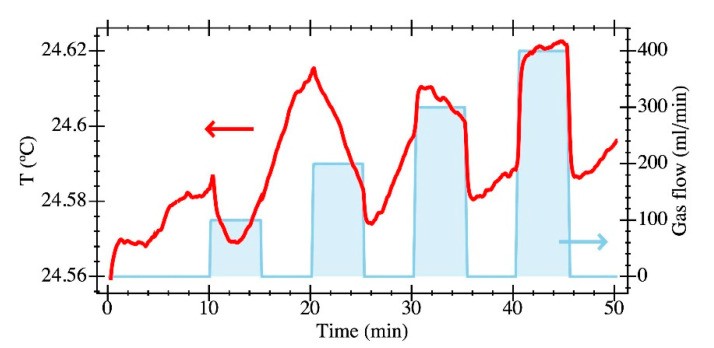
Time evolution of the temperature during the gas flow experiment measured into the sealed chamber (red line). The gas flow is also depicted in light blue.

**Figure 12:**
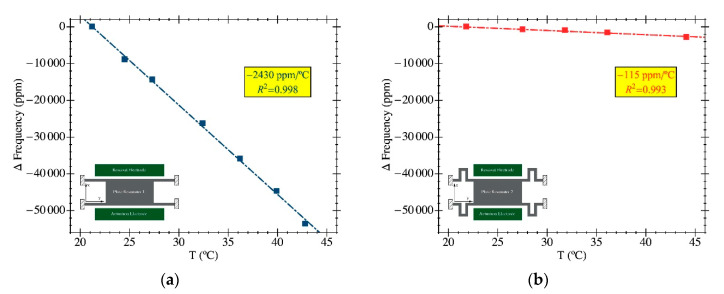
Resonant frequency dependence versus operation temperature calibrated into a climate chamber where the RH has been kept at 40 ± 1% for the whole curve. The DC bias voltage is 30 V for both resonators: (**a**) PR1; (**b**) PR2. Both plots preserve the same vertical scale in order to obtain a fairer visual comparison.

Temperature was carefully measured by including a thermal probe in the PCB design together with the IC connections to characterize its time evolution within the sealed chamber. Any gas delivery system is suitable for inducing local temperature variations caused by thermodynamic effects as a consequence of a gas expansion as a result of relative pressure changes or thermal differences between the gas supply and the resonator circuit. Figure 11 shows the thermal variations during one of the flow rate experiments, obtaining a temperature variation below 0.1 °C in the whole range. The impact of temperature on the resonant frequency was determined through a thermal calibration procedure obtained as in [31], where the IC was placed into a climate chamber with a constant relative humidity of 40% during a temperature sweep while acquiring the resonance frequency. Experimental results are given in Figure 12, from where the thermal sensitivities were computed to be −2430 ppm/°C for PR1 and −115 ppm/°C for PR2. The thermal insensitivity of PR2 vs. PR1 represents a dramatic improvement of more than one order of magnitude due to the folded flexure anchors. The temperature-related relative frequency change was 243 ppm in the worst case for PR1 and 11.5 ppm for PR2. These results conclude that the differences in frequency shifts experimentally measured during the gas flow trials were not due to temperature variations, and demonstrate that the PR2 design represents a significant advantage for a gas-sensing platform as it provides lower temperature variations. Interestingly, the measured temperature coefficient for PR2 was closer to the state-of-the-art results reported in [32] using the same technological approach, but with an active temperature compensation system that increased the overall power consumption.

### 4.4. Gas Flow-Induced Stationary Deflection

The reported experimental data support the notion that the root cause of the frequency drift induced by the gas flow was caused by the drag force exerted on the resonator, inducing its deflection. If the solid–fluid interaction is large enough to deflect the resonator out of equilibrium, it enters the non-linear stiffness region (see Section 3.2). If mechanical spring-hardening is the dominant mechanism (as happens for PR1 from Figure 4a), then when the structure is deflected by the fluid flow, its stiffness constant increases together with the resonance frequency, as shown in Figure 6a. However, if the dominant mechanism is not the spring-hardening (resonator PR2 in Figure 4b), then the resulting frequency shift after the gas stream is applied is much smaller when compared to PR1 (see Figure 6b). To thoroughly confirm these claims, one can relate the static deflection caused by the fluid drag force to the required change in the stiffness constant to provide a quantitative verification. For the PR1 structure, it is easy to find that the 1% change in resonance frequency (observed in Figure 6a) requires a 2% change of its stiffness constant. According to Equation (7) and the non-linear model in Figure 4, such an increase in the stiffness constant requires a resonator static deflection of 90 nm from the zero-deflection equilibrium. From Equation (5), and assuming a turbulent regime for the fluid flow, the drag force-induced deflection obtained is 30 nm, which is in the same order of magnitude as the computed value. Notice that an accurate estimation of the induced force is not feasible due to the turbulent nature of the fluid under test, and the non-linear model supported by COMSOL simulations might not provide full matching with the measured device because of fabrication stress, tolerance, etc.

## 5. Conclusions and Discussion

The reported experimental results highlight the impact of gas flow on the frequency shift of MEMS resonators caused by the fluid induced drag force without considering any chemical interaction. Such experimental data match perfectly with the analytical expressions. Our results show that the geometrical conditions of the structures supporting the resonant plate are capable of alleviating the disturbing effects of the solid–fluid interaction by reducing the spring-hardening effect as much as possible. Experimental data highlight that a calibration procedure of the overall experimental setups used for resonant gas sensors is mandatory to determine the final resolution of the overall system. The fabricated and measured CMOS-MEMS plate resonators specifically designed for gravimetric gas sensing monolithically integrated on-chip together with the readout amplifier operating as self-sustained oscillators show that the folded flexure anchor geometry mitigates this effect. The net result was a 5× reduction in the gas flow impact on the MEMS resonators’ frequency response conceived as mass distributed sensors for VOC monitoring. Moreover, we found the frequency shift to decrease with the fluid flow, i.e., the frequency variations were tinier for lower values of the flow rate only for the folded flexure geometry. A thorough analysis of the phenomena related to the resonance properties modified by the gas stream is supported by the experimental data of both under open-loop and closed-loop topologies.

Additionally, our work also shows that the folded flexure anchors provide a dramatic improvement temperature sensitivity close to 20× that of the already existing design, achieving −115 ppm/°C. Such a result is obtained without the need of adopting active temperature compensation schemes and using a single metal layer resonator to keep the mass sensitivity as high as possible [6].

The main contribution is the derivation of a method to diminish the impact of a gas course over the resonance properties of a MEMS resonator by anchor re-design with the consequent gain in overall gas sensitivity. Additionally, we proved that the very same shape also improves the temperature sensitivity of these resonators. Both aspects are key in the design of gas sensing structures since a transport system needs to be implemented for sensor calibration, thus inducing both temperature variations and gas flow impact.

## Figures and Tables

**Table 1:** Resonator fabrication parameters and computed mass sensitivity per unit area.

Resonator	Parameter	Symbol	Value
PR1	Beam length	$L_{b}$	10 μm
	Beam width	$W_{b}$	0.8 μm
	Thickness	t	0.85 μm
	Platform length	$L_{P}$	41 μm
	Platform width	$W_{P}$	10.2 μm
	Driver–resonator gap	s	0.6 μm
	Mass density ^1^	ρ	3000 kg·m^−3^
	Mass sensitivity per unit area	$S_{m,a}$	210 pg·Hz^−1^ cm^−2^
PR2	Beam length	$L_{b}$	4.6 μm
	Truss length	$L_{t}$	2.4 μm
	Beam width	$W_{b}$	0.8 μm
	Thickness	t	0.85 μm
	Platform length	$L_{P}$	41 μm
	Platform width	$W_{P}$	10.2 μm
	Driver–resonator gap	s	0.6 μm
	Mass density ^1^	ρ	3000 kg·m^−3^
	Mass sensitivity per unit area	$S_{m,a}$	270 pg·Hz^−1^ cm^−2^

^1^ The beam mass density was computed as a composite material as in previous works [6].
